# I Feel Like I Am Flying and Full of Life: Contemporary Dance for Parkinson’s Patients

**DOI:** 10.3389/fpsyg.2021.623721

**Published:** 2021-07-05

**Authors:** Anat Bar, Johanna Czamanski-Cohen, Judith Dita Federman

**Affiliations:** ^1^The School of Creative Arts Therapies, Faculty of Social Welfare and Health Sciences, University of Haifa, Haifa, Israel; ^2^The Emili Sagol Creative Arts Therapies Research Center, Faculty of Social Welfare and Health Sciences, University of Haifa, Haifa, Israel

**Keywords:** quality of life, psychological flexibility, group and interpersonal processes, Parkinson’s disease, contemporary dance

## Abstract

Parkinson’s is a neurodegenerative disease characterized by motor and non-motor symptoms which are strongly associated with patients’ quality of life, affecting social skills and support. It strikes not only the motor abilities but may harm cognitive and emotional functioning. For the past 15 years, contemporary dance has been employed as an intervention to help people diagnosed with Parkinson’s disease cope physically and mentally by way of motor, vestibular, and sensory stimulation as well as social interaction. In this study we aimed to examine psychological flexibility, creative self-efficacy and quality of life of Parkinson’s patients participating in contemporary dance sessions. To obtain this goal we conducted a cross-sectional comparative study of 50 Parkinson’s patients aged 50–87 years, half of which had been participating for at least 3 months once a week, in contemporary dance classes, and the matched controls participated in a verbal support group. Study participants completed questionnaires after participating in a dance class (Intervention) or in a support group (control). Participants in the intervention group were also asked to answer three open-ended questions that examined the experience of participating in contemporary dance classes. We found that psychological flexibility and quality of life were significantly higher in the dance class participants. Participants reported positive changes that occur in their overall feeling and quality of life following their participation in dance classes. Since PD patients’ experiences are deeply embedded in the body, it is significant to explore the use of movement in treatment. The importance of the study is in its potential to highlight the relationship between psychological flexibility and quality of life and to increase awareness of clinicians treating Parkinson’s patients to the importance of incorporating dance as an inherent part of a multidisciplinary team effort.

## Introduction

Parkinson’s disease (PD) is the second most prevalent neurodegenerative disease worldwide which affects the control of body movements, and has shown 2.4 times increase between 1990–2016, with 6.1 million individuals diagnosed with PD in 2016, and this number is on the rise (Armstrong and Okun, 2020). PD was first described by James Parkinson in 1817 and was coined “the shaking palsy.” The major symptoms have not changed since it was first described, and diagnosis is based on observed motor signs (Davie, 2008; Jankovic, 2008). In PD neurodegeneration is caused by a loss of dopaminergic neurons in the basal ganglia and the accumulation of α-synuclein, which can be found throughout the autonomic nervous system (olfactory bulbs, the vagus and the enteric plexus), and leads to cell death. The motor signs that lead to diagnosis only occur after there is over 70% dopaminergic loss in the substantia nigra. Thus, the neurodegeneration related to PD begins about 20 years before diagnosis (Kalia and Lang, 2016). The disease has an insidious onset and is characterized by both motor and non-motor symptoms. The major motor symptoms include stiffness/rigidity, unilateral tremor mainly at rest, slow movement (Bradykinesia), and postural instability. In addition to these physical difficulties, psychological, non-motor symptoms may include depression, lack of motivation, and anxiety, which are strongly associated with patients’ QoL (Davie, 2008; Jankovic, 2008). The array of symptoms as well as the rate of progression is unpredictable, but eventually PD patients become extremely limited, and need hands on assistance for daily living (Sveinbjornsdottir, 2016).

While the medications for the treatment of motor symptoms are inexpensive and lead to significant symptom reduction, this reduction can be temporary and the coping with on and off periods can be frustrating for PD patients (Connolly and Lang, 2014). Preventative treatment for PD has yet to be identified, however physical activity has been found to be a protective factor for PD (Reuter et al., 1999; Crizzle and Newhouse, 2006; Goodwin et al., 2008).

The motor and psychological symptoms of PD significantly affect the QoL of PD patients which deteriorates with disease exacerbation (Schrag et al., 2000a,b). Executive functions are also impaired in PD with beneficial and detrimental effects of dopaminergic medications. The executive dysfunctions are related to deficits in motor function and psychiatric symptoms (Dirnberger and Jahanshahi, 2013). Thus, PD treatment needs to be a multidisciplinary effort aimed at symptom reduction, rehabilitation and slowing the slope of deterioration. Thus, we need to find ways to improve motor and cognitive skills and achieve behavioral gains. An extensive review reported that physical activity could have significant preventative and rehabilitative properties for cognitive and brain function (Kramer and Erickson, 2007).

### Complementary Approaches to PD Treatment

As mentioned above, PD is a degenerative disease and medication have some effect that can be enhanced with rehabilitative interventions that focus on physical activity to reduce symptoms and improve (QoL) for PD patients (Goodwin et al., 2008). Exercise improves physical performance and daily activity in Parkinson’s patients (Reuter et al., 1999; Baatile et al., 2000; Goodwin et al., 2008; Ahlskog et al., 2011).

There are several physical and complementary approaches designed to reduce symptoms and improve QoL of PD patients. For example, active theater therapy, has been shown to improve the psychological well-being of Parkinson’s patients by reducing depression, apathy, anxiety, and even self-stigma with respect to a control group undergoing physical therapy (Modugno et al., 2010; Mirabella et al., 2017a). Furthermore, emotional theater training has obtained gains by possibly improving participants perception of their disease (Mirabella et al., 2017a). Studies have shown that skill-based activities that improve tempo-spatial accuracy along with aerobic exercise improves oxygen consumption to enhance circulating and respiratory efficiency and are beneficial in obtaining these goals. For example, in a randomized controlled trial (RCT) of 186 PD patients, multidisciplinary rehabilitation was shown to significantly reduce symptoms and improve QoL in the short and long term (Ferrazzoli et al., 2018). Dance for Parkinson’s patients is a way of incorporating the positive contribution of exercise to patients. It provides an opportunity to combine exercise and movement with rhythm, creativity and expression, along with changes and transitions between qualities and forms of movement, and the use of space and time. Above all, moving together with other people, talking and listening to music while moving, supporting and being supported, gives the power to cope with depression, anxiety, and lack of motivation so often seen in PD patients.

Dance therapy that utilized tango dancing (Hackney and Earhart, 2009b) and Irish dance (Volpe et al., 2013) have shown to improved balance and reduced frequency of falls. Tai chi is a form of martial arts that includes engaging in slow and focused movement has been shown to improve postural stability (Li et al., 2012), while a meta-analysis showed improvements in motor function and balance while, improvements in non-motor cognitive and affective symptoms were absent or very limited (Zhou et al., 2015).

### Contemporary Dance and PD

Contemporary dance was developed in the middle of the 20th century and has since grown to be one of the dominant styles in the world of professional dance. The style was initially influenced by classical ballet, modern dance and various jazz styles. In comparison to dance that requires following certain rules, such as planned changes of direction, accompanying the movement using gaze and specific dance steps, such as in Tango, the uniqueness of contemporary dance is the emphasis on improvisation, unexpected changes in rhythm, speed and direction, and the combination of multiple dance styles (McGrath and Meehan, 2017). Contemporary dancers focus on versatility, the use of gravity and working on the floor, which is often done barefoot and with different music styles (Albright, 2010; Laban and McCaw, 2011; McGrath and Meehan, 2017). The connection between contemporary dance and Parkinson’s was born out of the need to allow PD patients to experience pleasure that will enhance motivation for ongoing participation, of movement combined with creative expressive techniques (Butt, 2017), and dance for health (Bergman and Sabah Teicher, 2018).

Dancing has been shown to lead to significant improvements in physical symptoms like, rigidity, freezing, tremor, balance, gait, onset of movement, range of movements, motor ability, facial expressions and posture (Bunce, 2008; Hackney and Earhart, 2009a; Heiberger et al., 2011; Houston and McGill, 2013; Hashimoto et al., 2015; Bearss et al., 2017; De Natale et al., 2017; McRae et al., 2018). As well as, effect emotional symptoms like depression, anxiety, helplessness, the ability to feel joy and improve QoL (Marchant et al., 2010; Aguiar et al., 2016). Dancing benefits social communication as well (Heiberger et al., 2011).

Westheimer et al. (2015) showed the effect of attending 16 biweekly dance classes. Significant improvement was found in physical and emotional symptoms like gait and tremor, depression and increased social benefits. Bunce (2008) showed significantly improved coping skills, social interaction and decreased symptoms of emotional distress resulting from dance classes. A larger study reported significant improvement in functional mobility and self-efficacy in 61 PD patients that were related to QoL (McRae et al., 2018). Dance classes allow Parkinson’s patients physical activity (Westheimer et al., 2015), feelings of joy and freedom (Houston and McGill, 2013), boost self-confidence (Hackney and Bennett, 2014), discovering new movement options and reducing disease symptoms (Aguiar et al., 2016). Dancing in a group is a social activity that may increase social skills and reduce loneliness along with the above-mentioned benefits of the movement itself (Heiberger et al., 2011). The moments of interaction with other individuals engaged in dancing enables the learning of new movements by inspiration and encouragement from others (Fink et al., 2009). While the benefit of group dance for PD patients has been relatively well established, the mechanisms through which this benefit occurs and the ways in which dance has an effect that differs from other forms of physical activity have yet to be examined.

### Why Might Dance Help? Creativity and Flexibility

Creativity is the trait that underlies the act of creation and is historically defined as the creation of something new through a dynamic process of growth and development (Runco and Jaeger, 2012). Self-efficacy is a concept developed by Albert Bandura in 1995, defined as the individual’s belief in his abilities to achieve a particular goal or task (Bandura, 2010). Creative self-efficacy is defined as the individual’s perception of his ability to utilize creative thinking and execute these thoughts (Torrance and Safter, 1989). In other words, because a person feels creative, he will be more likely to be creative (Tierney and Farmer, 2002). Higher levels of creative self-efficacy increase the tendency to invest effort in performing the task even in the face of difficulties and obstacles. Creative self-efficacy is related to motivation and creative performance in students (Beghetto, 2010), as well as efficiency, achievement, and interpersonal skills in the workplace (Mathisen, 2011).

Psychological flexibility (Kashdan and Rottenberg, 2010) is defined as the ability to adapt to different situations by redefining and shifting mental resources. Psychological flexibility is dependent on one’s awareness, ability to recognize shifting situations and being open to adapt to different situations. Individuals who have lower levels of psychological flexibility as measured by the Acceptance and Action Questionnaire (AAQ) have higher anxiety and depression, as well as lower levels of daily functioning (Bond et al., 2006, 2011). An intervention designed to increase psychological flexibility improved the emotional and psychological well-being of participants (Fledderus et al., 2010).

### Dance, Creativity, and Psychological Flexibility

Dance is a creative activity that allows new and original processes to take place, and may enhance psychological flexibility (Hui et al., 2008) and adaptation to changing situations (Kashdan and Rottenberg, 2010). The psychological profile of experienced dancers found that dancers have a collection of dominant personality traits such as creativity, self-confidence, high mental skills, self-awareness, ability to change and accept change (Taylor and Estanol, 2015). Alter (2004) postulated that the physical flexibility required of dancers, contributes to mental flexibility while enhancing a dancer’s motivation and ability to improve their physical performance.

Creative thinking requires originality and flexibility, and it makes sense to believe that creativity and flexibility may assist PD patients to cope with the cognitive decline and degeneration of motor skills. However, there are very few studies that examine the relationship between PD and creativity and psychological flexibility. A review that examined the relationship between PD and artistic creativity, examined 40 artworks (pictures, sculptures, novels and poetry) of PD patients and found that patients taking dopaminergic medications had higher artistic skills and motor control, as well as better QoL, self-perception, and daily functioning (Inzelberg, 2013). In addition, a group of researchers postulated that patients receiving dopamine agonists for PD, may have increased artistic creativity as a side effect of dopaminergic therapy. They found that PD patients treated with dopamine agonists and/or levodopa had enhanced verbal and visual creativity as compared to healthy controls. The researchers suggest that dopaminergic agents may reduce latent inhibition leading to increased creativity (Faust-Socher et al., 2014). As mentioned, a hallmark of PD is a deficit in inhibitory control. The impairment of this executive function is known to influence behavioral and psychological flexibility (Dirnberger and Jahanshahi, 2013; Mirabella et al., 2017b).

Thus, the aim of the present study is to examine the relationship between psychological flexibility, creative self-efficacy and QoL in PD patients participating in contemporary dance classes. Furthermore, we would like to examine the experience of PD patients participating in dance classes. We hypothesize we will find that: A. The psychological flexibility among PD patients participating in contemporary dance classes will be higher than non-dancing controls. B. Creative self-efficacy among PD patients participating in contemporary dance classes will be higher than in non-dancing controls. C. The QoL among PD patients participating in contemporary dance classes will be higher than non-dancing controls.

## Materials and Methods

In order to obtain our study goals, we conducted a comparative community-based cross-sectional comparative study to examine the difference in the level of psychological flexibility, creative self-efficacy and QoL among PD patients participating in contemporary dance classes to a matched control group of PD patients attending a verbal support group. Since this study was conducted in a community setting, we based the match on age group. While we are aware that clinical studies often match samples based on level of functioning using Hoehn & Yahr status, Mini-Mental State evaluation, dopaminergic therapy expressed in Levodopa Equivalent Dose (LEDD), and/or the Unified Parkinson’s Disease Rating Scale (UPDRS III), being a community-based study, we did not have access to participants medical information, to include, disease stage, medical treatment or on off status, nor were we able to conduct diagnostic observations. Furthermore, enabling anyone to participate in dance classes at Yassmin Goddar is part of their philosophy, so groups are not divided by disease stage and participants are not asked to share details about their disease or treatment. We also asked participants to describe their experience aimed at examining the “lived experience” of participating in contemporary dance classes (Denzin, 1983).

### Procedure

The study participants were 50 PD patients (25 in the intervention group) aged 50–87 years (see Table 1 for demographics), who have been participating for at least 3 months in contemporary dance classes or a verbal support group. Prior to obtaining informed consent, all study participants received a written and verbal explanation of the study and its objectives. Participants in the contemporary dance group participated in contemporary dance classes at the “Yasmeen Godder” studio in Tel Aviv-Yafo. The guiding principle of contemporary dance classes in the studio is the focus on what promotes health. The lessons are based on the belief in the human ability to develop through dance and art (Bergman and Sabah Teicher, 2018). The participants are referred to as dancers, the content of classes is diverse and based on a supportive circle of teaching staff and volunteers. All sessions begin with a physical warm up in a circle consisting of moving body parts and joints, then while accompanied with music, the group leader starts with a free movement and invites participants to join in their own interpretation of the leader’s movement. As the group develops, participants are invited to enter the circle and continue the movement in a dyadic form with the leader in a back-and-forth mirroring and further developing their joint dance, all the while the rest of the group continues to mirror the movements of the dyad. Toward the end of the session all members rejoin the circle for a gradual goodbye that takes form by moving toward the middle of the circle and getting closer to one another and moving outward and gaining more space between the members.

**TABLE 1:** Descriptive and demographic characteristics of the study sample.

		Intervention group M(±SD)	Control group M(±SD)	*t*(df)	Total sample M(±SD)
Age (years)	87–50	69.44(7.76)	73.24(8.04)	−*1.70*(*48*)	71.34(8.05)
Length of disease (years)	1–31	11.24(6.59)	9.20(5.21)	1.21(48)	10.22(5.97)
		N(%)	N(%)	Z	N(%)
Gender	Male	4(16.0)	10(40.0)	1.89	14(28.0)
	Female	21(84.0)	15(60.0)		36(72.0)
Marital Status	Married	17(68.0)	18(72.0)	0.31	35(70.0)
	Not married (single, divorced, widow)	8(32.0)	7(28.0)		15(30.0)
Education	High school or less	10(40.0)	17(68.0)	1.99**	27(54.0)
	Academic (BA, MA, PhD)	15(60.0)	8(32.0)		23(46.0)
Experience with dance	Yes	12(48.0)	7(28.0)	1.46	19(38.0)
Experience with ballroom dance	Yes	9(36)	1(4)	3.024***	10(20.0)
Sports activities	Yes	23(0.97)	21(1.07)	0.41	44(88.0)
Physical Therapy	Yes	10(0.5)	18(0.46)	−2.36*	28(56.0)

*p = 0.023, **p = 0.025, and ***p = 0.005.

The non-dancing control group was contacted through several professional organizations that provide support groups for PD patients. These groups entail sitting in a circle with a group leader who provides an environment in which members take turn expressing difficulties related to coping with PD and the leader and members provide support.

Participants who expressed their consent to participate in the study and signed an informed consent form were asked to complete questionnaires at the end of dance class or support group. Participants in the dance classes were also presented with open-ended questions regarding their experience, as detailed below.

### Questionnaires

The demographic questionnaire includes reference to age, sex, education, marital status, time of diagnosis of the disease, past and present movement or dance experience.

*Psychological flexibility* was measured with the Acceptance and Action Questionnaire (AAQ) (Hayes et al., 2004) which measures experiential avoidance and psychological inflexibility. The scale contains seven items with a range of 1–7 and is scored by averaging the items so that higher scores indicate higher psychological flexibility. The items deal with negative assessments of emotions, avoidance of thoughts and feelings, and behavioral adjustment in the presence of difficult thoughts or feelings (Bond et al., 2011). The AAQ has been used as a research tool in more than 30 studies and has been found to be related to levels of depression, anxiety, general mental health, job satisfaction, future job loss, and future job performance (Hayes et al., 2006; Chawla and Ostafin, 2007). Cronbach alpha is 0.84, and the retest reliability for 3 and 12 months is 0.81 and 0.79, respectively (Bond et al., 2011). In the present study, Cronbach α = 0.92.

*Creative self-efficacy* was measured using the Creative self-efficacy questionnaire (Tierney and Farmer, 2002) designed to measure creative performance in a work environment. The questionnaire includes four statements, and participants are asked to rate on a scale of 1 to 7 the extent to which they feel that each statement describes them. The index was calculated using the average of the items in such a way that a higher average indicates greater self-efficacy. The questionnaire has Cronbach alpha of 0.83 and has been widely utilized in studies (Shalley et al., 2004; Shin and Zhou, 2007; Gong et al., 2009). In the present study, the questionnaire was found to have high internal consistency of α = 0.87.

*Quality of life* was measured with the *Parkinson’s Disease Questionnaire (PQD)* which is the most commonly used tool to assess QoL among Parkinson’s patients and assesses eight different domains: such as mobility, social support, communication and physical discomfort, on a scale of 1 to 5. The indices are calculated by averaging the items, and higher scores indicate a better QoL. The questionnaire has two versions: a 39-question version and an abbreviated 8-question version (Peto et al., 1995). Internal reliability of the questionnaire ranges from 0.64–0.95. It has been translated into many languages, as well as translated and validated in Hebrew (Moore et al., 2005). Participants in the present study answered the abbreviated questionnaire, as well as four additional questions representing emotional wellbeing from the full questionnaire. In our study the questionnaire had high internal consistency for the overall QoL index Cronbach α = 0.86, as well as the emotional QoL index Cronbach α = 0.83.

*The open-ended questions* examined the experience of participating in dance classes, the perception of the QoL and the perception of the impact of dance on the lives of the participants. Participants were asked to write about how they felt before and/or after attending a contemporary dance class, the impact of the dance classes on their daily life and QoL. The choice of a combination of open-ended questions in the study was intended to expand the information that is attainable in self-report measures and to obtain the subjective experience of dance class participants

### Data Analysis

Quantitative data analysis was performed using SPSS version 25 (IBM Co., Armonk, NY, United Sates). Participants’ background characteristics were described using means and standard deviations for continuous variables (age, duration of illness), and using frequencies and percentages for non-continuous variables (gender, education). Differences between the groups were examined using a *t*-test for the continuous variables and using a Z analysis to compare proportions between two samples for the non-continuous variables. Internal consistency was calculated for all study variables (Cronbach α), and the variables were defined using the item averages. Averages and standard deviations for the study variables were calculated and described, as well as Pearson correlations between them with respect to the whole sample. Subsequently, correlations between the study variables and the background variables were examined in order to examine the need to monitor any background variables when examining the research hypotheses. The hypotheses were examined using a multivariate analysis of variance while controlling for previous participation in movement and conducting any sporting activity (MANCOVA).

The qualitative data was analyzed using thematic analysis to examine the meaning of the data obtained along with a holistic description of the participant’s experiences. In the process of analyzing the texts, the respondents “answers were grouped into key themes related to the participants” experience in the lessons and the sense of the impact of this experience on their lives. In the first stage, the first author read each answer to obtain a first impression of the main topics, in the second stage, we read each questionnaire again while performing open coding in order to organize the content into main content categories (Creswell, 2014).

### Ethical Considerations

The study received the approval of the Ethics Committee of the Faculty of Social Welfare and Health Sciences at the University of Haifa (Approval No. 241/18). All study participants received a written and verbal explanation of the study and its objectives and agreed to participate in it with the understanding that they are not obligated to answer all questions and can stop participating at any time.

## Results

We were able to recruit 50 participants for our study (25 in the intervention group). There were no statistically significant differences in the demographic characteristics between the study and control group participants. The participants had been diagnosed for an average of 10 years with 72% women, most of the participants married (70%), and close to half had an academic education (46%) (see Table 1 for demographics and descriptive statistics). Out of the 25 participants in the intervention group, 36% also participate in ballroom dance classes. Twenty-one participants from the intervention group (84%) and 16 from the comparison group (64%) take part in some sports classes (swimming, gymnastics, yoga, etc.) with no significant difference between the groups (*Z* = 1.61, *p* = 0.107). Ten participants from the intervention group (40%) and 18 from the comparison group (72%) received physical therapy, a difference which was found to be significant between the groups (*Z* = 2.28, *p* = 0.023).

We examined the relationship between the study variables in the entire sample, and found positive, significant correlations between psychological flexibility and perception of QoL (see Table 2). When examining the means and standard deviations of study variables, we found relatively high levels of creative self-efficacy, psychological flexibility and QoL (see Table 2). The distribution of study variables was normal (Skewness = −0.47 to −0.88, SE = 0.34).

**TABLE 2:** averages and standard deviations and correlations between study variables (*N* = 50).

Variable (range)	*M* (±*SD*)	Creative self-efficacy	QoL- General scale	Emotional QoL
				
Psychological flexibility	5.43 (1.51)	0.15	0.74***	0.62***
(1–7)				
Creative self-efficacy	4.97 (1.36)		0.25	0.28
(1–7)				
QoL- General scale (1–5)	3.68 (0.84)			0.82***
Emotional QoL (1–5)	3.96 (0.84)			

***p = 0.0001.

Correlations were calculated between the study variables and all the background variables to examine the need for statistical control of the background variables when examining the research hypotheses. The age and duration of the disease are continuous variables, while the other background variables were defined as dichotomous variables: gender (1-men, 0-women); Marital status (1-married, 0-unmarried); Education (1-academic, 0-to high school/high school); Previous participation in the movement; Participation in physical therapy; Any sporting activity (1-yes, 0-no).

We examined the relationship between the demographic and study variables and found no association between age, gender, marital status, education, duration of illness, or previous background in dance or sports and psychological flexibility, self-efficacy, or general or emotional QoL. Thus, the research hypotheses were examined controlling for previous participation in movement and sports activities.

The three hypotheses were tested using a multivariate variance analysis (MANCOVA) of the study variables, by group, controlling for previous participation in movement and sports activities (see Table 3). In accordance with our first hypothesis we found that psychological flexibility was higher among PD patients participating in contemporary dance classes 5.80(±1.35) in comparison to participants in the control group 5.06 (1.60) with a main effect of *F*(1, 46) = 4.75, *p* = 0.004. We also found higher general QoL in the participants of the dance group 3.98 (± 0.70) in comparison to the control group 3.37 (± 0.88) with a main effect of *F*(1, 46) = 6.66, *p* = 0.009. Finally, we found higher emotional QoL among participants in the dance group 4.20 (±0.75) in comparison to the control group 3.72 (±0.87) *F*(1, 46) = 4.12, *p* = 0.04. We did not find a statistically significant difference in creative self-efficacy between the dance group 5.27 (± 1.25) and the control group 4.67 (±1.42). Thus, the first and third research hypotheses were confirmed, and the second research hypothesis was not confirmed.

**TABLE 3:** Means, standard deviations and F values of the study variables, by group *N* = 50.

	Dance group *M* (*SD*)	Control group *M* (*SD*)	*F*(1,46) (η^2^)
Psychological flexibility	5.80 (1.35)	5.06 (1.60)	4.75*(0.090)
Creative self-efficacy	5.27 (1.25)	4.67 (1.42)	1.08(0.023)
QoL- General	3.98 (0.70)	3.37 (0.88)	6.66**(0.127)
QoL- Emotional	4.20 (0.75)	3.72 (0.87)	4.12*(0.082)

*p < 0.05 and ***p < 0.001.

The dancing participants wrote about their sensations and experience before, during and after participating in dance sessions, and several themes were identified. The first theme, “Physical and emotional sensations” pertains to physical and emotional changes that were identified after participating in dance classes. This theme can be further divided into sub-themes of difficulties (weakness, poor walking, instability, fear, and uncertainty) versus benefits (improved balance, pleasure joy and self-confidence). More specifically, as Mike (pseudo name) stated: “I feel a little “off” before class, I have difficulty walking, difficulty speaking and balance problems but the pleasure following the dance sessions and meeting with the dancers and all the people in the group makes me feel joy and in a great mood”; John said: “Before class I feel general weakness especially in my calves”; Suzy said, before class—“I’m afraid I won’t be able to participate and dance, but at the end of the class I always say to myself: Wow, I succeeded, it was wonderful!.” Nicholas shared “My mood always improves after dancing, I go out in a great mood happy, bursting with adrenaline. I feel like I’m flying and full of life.” Nathan stated that he arrived at the studio with his cane, and when he left realized he forgot his cane in the studio.

The second theme “Changes outside the dance studio” pertains to changes that participants feel occurred in their lives following dancing. This theme is further broken down into subthemes of confidence (self-acceptance, changes in posture and movement in space), energy levels and increased social interaction. For example, Allan stated: “Dancing gives me confidence and changes my posture, movement and daily life when interacting with people. I have freed myself from many restraints and opened an important window in my life, to dare much more and believe in my abilities.” Marsha stated that dancing “gets me energized to do things, be happier. Dance adds joy, self-acceptance without criticism, physical flexibility and a great mood, I feel young and beautiful!.”

The third theme “My life without dance” pertains to ways in which participants described their experience before dancing or how their life might be if they did not dance. Sub themes in this category describes closedness versus openness, depression versus joy, repression versus awareness, rigidity versus flexibility, boredom versus joy, identity as patients versus dancers. For example, Joseph shared: “Without movement I lived a life of desolation, without the dance lessons I was much more closed and depressed.” Ruth explained: “The body and mind awareness I developed since I started dance sessions have become a part of me. Without dance I would have felt less joy in life, less ability to move my body, without the dance experience I would have been bored.” Linda concluded “Here there are no PD patients, but only dancers.”

In conclusion, the qualitative portion of our study strengthens the quantitative findings regarding the flexibility of the participants in dance classes, as well as of QoL that were found to be significantly better than in the comparison group and clarified their positive experiences and its impact on their everyday lives. However, creative self-efficacy was not found to be higher in the intervention group nor was this mentioned in the qualitative part.

## Discussion

The aim of the study was to examine psychological flexibility, creative self-efficacy and perception of QoL in PD patients who participated in contemporary dance classes for PD patients. Contemporary dance has been recognized as an auxiliary treatment for PD (Earhart, 2009). Dance classes allow PD patients physical closeness with others (Westheimer et al., 2015), feelings of joy and freedom (Houston and McGill, 2013), self-confidence (Hackney and Bennett, 2014), and the opportunity to discover new movement repertoire and a decrease in disease symptoms (Aguiar et al., 2016).

The findings of the current study point to significant differences in psychological flexibility and perception of QoL between the groups, while there was only a tendency for creative self-efficacy. Creativity in general has been found to be enhanced in PD patients taking dopaminergic medication (Faust-Socher et al., 2014). The qualitative portion of the study clarified the positive experiences of the participants in the dance classes, as they expressed positive changes in their felt QoL. While benefits of dance, such as Tango, have been shown in previous studies (i.e., Bunce, 2008; Hackney and Earhart, 2009b) contemporary dance provided the opportunity to examine a movement experience that emphasized free movement that potentially may have different benefits than structured dance. Thus, this experience provided the opportunity to begin to observe the relationship between psychological flexibility, creative self-efficacy and QoL which has been lacking in studies about dance and PD.

As hypothesized, the level of psychological flexibility was found to be significantly higher in the intervention group. This finding can be explained by the many complex stimuli and movement changes during the lessons that require flexibility and concentration. PD patients, practice starting and stopping, changing direction, during the dance. Pleasure is not just an added value but enhances motivation to keep practicing in response to several stimuli simultaneously. Psychological flexibility allows a person to adapt to environmental changes and redefine his or her physical and mental abilities, and to adapt to new realities (Kashdan and Rottenberg, 2010). The ability to adapt to a changing reality has an effect on QoL and is especially relevant for PD patients whose functioning changes at an unpredictable pace and pattern. The study findings did not indicate a significant difference for creative self-efficacy, although the mean level was higher among the dancers. Dance offers the opportunity to improvise and allows for creative and personal expression (Albright, 2010; McGrath and Meehan, 2017). Creative self-efficacy may develop as a result of creative efforts (Tierney and Farmer, 2002). However, as mentioned earlier it has been postulated that creativity is enhanced as a result of dopaminergic therapies in PD patients (Faust-Socher et al., 2014).

QoL among PD patients participating in dance classes was higher than in non-dancers, which has been established in previous studies (Houston and McGill, 2013; Bearss et al., 2017; McRae et al., 2018). One of the current study aims was to examine the relationship between psychological flexibility and QoL, which has been supported here. Improved QoL among participants in the dance group can be possibly explained by the satisfaction achieved following succeeding to move together with others, in a group in a way that is in contrast to symptoms of the disease (Westheimer et al., 2015).

The group framework of the dance sessions helps to alleviate feelings of loneliness (Davie, 2008). Participants watch others while dancing and are being seen themselves. This enables the participants to feel connected to the other dancers in the group. Moving together with other people, supporting and being supported, gives PD patients the strength to cope with depression, anxiety and helplessness (Houston, 2011). In dance classes for PD patients, group inventions lead to an experience of empowerment while self-esteem and recognition of their ability to cope with the surrounding reality is strengthened (Chaiklin, 2015). The added benefit of dancing in a group for psychological flexibility and improved QoL should be noted as our control group also had group interactions.

Participants described the impact of dance on their daily lives in a positive way, describing a sense of liberation, being filled with confidence and belief in abilities, greater flexibility, self-acceptance and a sense of joy and happiness. Most participants addressed the improved QoL, and most described positive, physical and/or social, emotional changes related to self-satisfaction, sense of accomplishment and added value to their lives, as mentioned in the literature (Quiroga-Murcia et al., 2010; Houston and McGill, 2013; Bearss et al., 2017; Bergman and Sabah Teicher, 2018; McRae et al., 2018). We can make this conclusion regarding participation in contemporary dance classes but can not generalize these findings to different and more structured types of dance classes.

## Limitations and Conclusion

The aim of the present study was to examine psychological flexibility, creative self-efficacy and QoL of PD patients participating in dance sessions. This study led to an improvement of our understanding of psychological flexibility and its relationship to QoL in PD patients who participate in dance sessions, but it is not without limitations. A main limitation is that this study did not randomize participants to an intervention and control group. There is a possibility that those who choose dance classes have higher levels of creative self-efficacy, psychological flexibility and a better QoL to begin with. Thirty six percent of the participants in the contemporary dance classes also participated in ballroom dancing. Besides the fact that this is a cross- sectional comparison study, this further limits our ability to conclude that the differences between the groups stem from the contemporary dance experience. Furthermore, since this is a community-based study, we did not have access to medical information such as Hoehn & Yahr status, Mini-Mental State evaluation, dopaminergic therapy expressed in Levodopa Equivalent Dose (LEDD), UPDRS III or were we able to match controls according to this or to their on off stage in medical treatment. In addition, information is missing regarding the emotional state of the subjects prior to the interventions, thus we cannot conclude that the higher levels of psychological flexibility and QoL are a result of participation in contemporary dance sessions. In addition, there is a wide range of symptoms in PD patients and their severity varies as well, making it difficult to conduct an accurate between subject comparison. Participants in this study had full understanding of the study aims and objectives following filling out informed consent, and participants may be biased toward the dancing doing them good, as they have chosen to participate in dance classes. Another limitation of the current study lies in the difficulty of PD patients to fill out questionnaires. While participants expressed the motivation to cooperate, they have had physical or cognitive difficulties filling out the questionnaires.

This study has clinical relevance and significance as it brings the relationship between psychological flexibility and QoL into awareness, thus may encourage clinicians working with PD patients to give focus on developing psychological flexibility. Future research may want to take our study further by assessing changes across time with PD patients who have not yet experienced dance for PD sessions before and are randomized to a control group within a medical setting with full access to medical information. In addition, while dance is generally recommended by physicians working with PD patients, it would be useful to examine whether dance for PD is something that should be encouraged in individuals who do not have an interest in dance from the outset. Other recommended studies can be those examining differences in the benefit of different types of movement-based interventions. Further questions for continued research, are, to closely examine the relationship between psychological and physical flexibility, thus possibly enabling an additional pathway to counter-effect the symptoms of degenerative disease. Since research with individuals coping with degenerative diseases is very challenging, our small study serves as an additional scaffold supporting the implementation of dance as an inherent part of a multidisciplinary approach to PD treatment.

## Data Availability Statement

The raw data supporting the conclusions of this article will be made available by the authors, without undue reservation.

## Ethics Statement

The studies involving human participants were reviewed and approved by the Ethics Committee of the Faculty of Social Welfare and Health Sciences at the University of Haifa (Approval No. 241/18). The patients/participants provided their written informed consent to participate in this study.

## Author Contributions

AB collected the data and participated in the data analysis as part of her MA thesis. JC-C and JF co-wrote the manuscript and participated in the data analysis. All authors contributed to the article and approved the submitted version.

## Conflict of Interest

The authors declare that the research was conducted in the absence of any commercial or financial relationships that could be construed as a potential conflict of interest.
